# Prevalence of *Helicobacter pylori vacA*, * cagA*, and *iceA* Genotypes in Cuban Patients with Upper Gastrointestinal Diseases

**DOI:** 10.1155/2015/753710

**Published:** 2015-04-06

**Authors:** Onelkis Feliciano, Oderay Gutierrez, Lidunka Valdés, Trini Fragoso, Ana Maria Calderin, Antonio Eduardo Valdes, Rafael Llanes

**Affiliations:** ^1^Microbiology and Clinical Branches, Tropical Medicine Institute “Pedro Kourí”, Avenida Novia del Mediodía Km 6 1/2, P.O. Box 601, Marianao 13, Havana, Cuba; ^2^Endoscopy Department, Pedro Borrás Hospital, Havana, Cuba

## Abstract

Virulence factors of* Helicobacter pylori* can predict the development of different gastroduodenal diseases. There are scarce reports in Cuba about* H. pylori* isolates genotyping. The aim of the present investigation was to identify allelic variation of the virulence genes* vacA*,* cagA*, and* iceA *in sixty-eight patients diagnosed as* H. pylori* positive by culture. In seven out of 68 patients, strains from both gastric regions were obtained and considered independent. DNA was extracted from all the* H. pylori* strains and evaluated by PCR-genotyping. The* vacA* s1 allele,* cagA* gene, and* iceA2* allele were the most prevalent (72.0%, 56.0%, and 57.3%, respectively). Alleles from m-region showed a similar frequency as s1a and s1b subtypes. The presence of multiple* H. pylori* genotypes in a single biopsy and two gastric region specimens were found. Significant statistical association was observed between* iceA2* allele and patients with non-peptic ulcer dyspepsia (NUD) (*P* = 0.037) as well as virulence genotypes (s1,* s1m2*) and patients over 40 years old (*P* < 0.05). In conclusion, the results demonstrated a high prevalence of* H. pylori* virulent genotypes in Cuban patients over 40 years old while* iceA2* alleles demonstrated a good specificity in patients with NUD.

## 1. Introduction

*Helicobacter pylori *is associated with the development of chronic gastritis, peptic ulcer disease (PUD), and gastric cancer (GC). Hence, since 1994, the World Health Organization has classified it as class I carcinogen [1]. Interestingly, despite the high prevalence of* H. pylori *infection in some countries, the frequency of severe diseases is much lower than other populations. In addition to host factors and diet, the varying outcomes of* H. pylori *infection could be related to the virulence of* H. pylori *strains differences [2].

Different virulence factors that play a role in the pathogenesis of the disease such as cytotoxin-associated gene A (CagA), vacuolating cytotoxin A (VacA), and* iceA* gene have been described [2, 3]. The* cagA *gene, which encoded the CagA protein, is reported to be found in more than half of the* H. pylori *isolates. It is known that* cagA *is a marker for the cag pathogenicity island and is associated with increased IL-8 production, nuclear factor-kB activation, mucosal inflammation, and development of PUD and GC [3].

The protein VacA is responsible for the gastric epithelial erosion observed in infected hosts. The* vacA* gene encoding the vacuolating toxin comprises three variable parts, the s-region (encoding the signal peptide) and two alleles, s1 and s2. Within the s1 allele, several subtypes (s1a, s1b, and s1c) can be distinguished. For the m-region (middle), two alleles, m1 and m2, have been recognized [4]. The VacA activity level is defined by* vacA* s- and m-regions combination;* s1m1* produces high amount of toxin and is considered the most virulent; however,* s2m2* produces an inactive toxin [4, 5]. In Western countries, infection with* vacA s1m1* strain is more common in patients with PUD than those with chronic gastritis [5]. Recently, a third polymorphic determinant of vacuolating activity (located in the middle of s- and m-regions) has been described as an intermediate (i) region [6].

The* iceA* gene has two alleles:* iceA1* and* iceA2*. The* iceA1* allele, encoding a CATG-specific restriction endonuclease, is regulated by the contact of* H. pylori* with the human gastric cells [7]. In Western countries the presence of* iceA1* allele is strongly associated with PUD [7, 8].

In Cuba, there are scarce investigations regarding the pattern of virulence genes in* H. pylori* strains [9, 10], but none have examined the s1 allele subtypes of* vacA* gene, the* iceA *gene nor the* H. pylori* strains genotypes isolated from younger or older patients. The aim of this study was to investigate the prevalence of* cagA*,* vacA,* and* iceA* genotypes of* H. pylori* isolates recovered from Cuban patients with dyspepsia.

## 2. Materials and Methods

### 2.1. Patients

Gastric biopsies from 150 patients referred to gastroscopy at two Cuban hospitals in Havana from 2009 to 2010 were collected. Patients with a history of gastric surgery, active gastrointestinal bleeding or who had received antibiotics, proton pump inhibitors, or bismuth compounds in the last four weeks were excluded. Sixty-eight patients (35 male and 33 female) with a mean age of 39.2 (years range = 9 to 68) reported positive for* H. pylori* infection by culture were included. The protocol was approved by the Ethical Review Committee of the Tropical Medicine Institute “Pedro Kourí” (IPK) and all patients provided an informed consent.

### 2.2. Culture and Genomic DNA Isolation

Antrum and corpus biopsy specimens from each patient were kept in sterile saline solution (0.9%) at 4°C. The endoscopic biopsy specimens were smeared on the surface of Columbia chocolate agar plates enriched with Dent supplement (Oxoid, England) and 1% of fetal calf serum (Gibco, USA) and incubated under microaerophilic conditions (Campy Packs, Oxoid) for up to 3 days.* H. pylori* isolates were identified by typical Gram-staining morphology and positive biochemical urease, oxidase, and catalase [11]. Biopsies of 68 patients yielded 75* H. pylori* isolates; those obtained from two gastric biopsy sites of seven patients were considered independent. Primary cultures of* H. pylori* were conserved at −80°C in brain heart infusion with 20% glycerol and later on were subcultured as it was described above.

All colonies from the subculture were used for chromosomal DNA extraction by Wizard Genomic DNA Purification Kit (Promega, USA) according to the manufacturer's instructions. DNA content and purity were determined by measuring the absorbance at 260–280 nm (Spectrophotometer MRC, Spain) and by amplification of* ureA* gene [12]. Samples were stored at −20°C before polymerase chain reaction (PCR) amplification was performed.

### 2.3. PCR

Primers used in this study are shown in Table 1. Amplification of* ureA*,* cagA*,* vacA,* and* iceA* genes by PCR was made in a volume of 50 *μ*L containing 1X PCR buffer (pH = 7), 3 mM MgCl_2_, 0.2 mM deoxynucleoside triphosphate, 0.5U Taq polymerase (Sigma, USA), 25 pmol of each primer, 2 *μ*L of chromosomal DNA, and sterile distilled water (Sigma). PCR amplifications were performed in an automated thermal cycler (Techne, Belgium). All runs included a negative DNA control consisting of PCR grade water and two positive DNA controls from* H. pylori* reference strains, ATCC43504 (genotype* vacAs1am1/cagA+/iceA1*) and 26695 (genotype* vacAs1bm1/cagA+/iceA1*). The PCR products were electrophoresed on 2% agarose gel stained with ethidium bromide (Promega) and visualized under UV light. Standards of 100 bp DNA Step Ladder (Promega) were used as molecular size marker.

### 2.4. Sequencing of s-Region

To analyze nucleotide sequence similarity of* vacA* genotypes among nontypable* H. pylori* strains for s1 region, entire s-region of* vacA* gene was amplified using both the forward and reverse primers (Table 1). Amplified products purified by High Pure PCR Product Purification Kit (Roche, Switzerland) were directly sequenced (Beckman Coulter, Belgium) using DTCS Quick Start Master Mix (GenomeLab, USA). Sequence comparison was carried out using the BLASTn program and the GenBank databases (http://blast.ncbi.nlm.nih.gov/Blast.cgi).

### 2.5. Statistical Analysis

Data were analysed using Chi-square test. Any *P* value < 0.05 was considered to be statistically significant. Association between clinical findings and genotypes was tested independently in two groups, defined according to age group (patients aged less than 40 years were grouped in group 1 and those within 40 years and older in group 2).

## 3. Results

### 3.1. Distribution of* vacA*,* cagA*, and* iceA* Genotypes

The* vacA* s-region was amplified in all 75* H. pylori* strains studied: 54 (72.0%) were identified as s1 and 17 (22.7%) as s2 and the remainder 4 strains (5.3%) harboured both alleles (Table 2). The s1 variants were detected in 94.4% (51/54 strains) and three strains could not be typed by the primers used in this study. In 49 of 51 strains only two single s1 subtypes were identified (25 as s1a and 24 as s1b) and two strains were classified as s1a-s1b subtype. Two of the three selected strains for sequencing the s1 region were identified as s1a-s1b variant with more than 91% of homology (IPK56C, IPK191C) and as s1a variant with 89% of homology (IPK201A) (GenBank accession numbers: KP462879, KP462880, and KP462878). All strains were typed by* vacA* m-region, resulting in 50.7% (38 strains) with m2 allele and 49.3% (37 strains) with m1 allele. Four possible single combinations of the s/m alleles were detected and the most frequent was* s1m1* (36 strains, 48.0%) followed by* s1m2* (18 strains, 24.0%),* s2m2* (16 strains, 21.4%), and* s2m1* (1 strain, 1.3%). Four strains (5.3%) corresponded with* s1-s2m2* combination. According to s1 subtypes (54 strains) the most frequent combination was* s1bm1* (19 strains, 35.2%),* s1am1* (15 strains, 27.8%),* s1am2 *(12 strains, 22.2%),* s1a-s1bm1* (2 strains, 3.7%), and* s1bm2* (1 strain, 1.9%).

The* cagA* gene was detected in 42 (56.0%) strains. Overall 68 (90.7%) isolates had a single* iceA* allele;* iceA2* was detected in 43 (57.3%) and* iceA1* in 25 (33.3%) strains. Seven strains were identified with both* iceA *alleles (7 strains, 9.3%).

### 3.2. *H. pylori* Genotypes and Clinical Association

Regarding endoscopy aspects of the mucosa, patients were distributed into non-peptic ulcer dyspepsia (NUD) in 64.7% (44/68), PUD in 35.3% (24/68: 10 gastric and 14 duodenal ulcers). Despite the fact that s1 allele of* vacA* gene and* cagA*+* H. pylori* strains were more frequently identified in patients with NUD (31 patients, 70.5%) and with PUD (16 patients, 66.7%), respectively, no statistical association was observed. However, the presence of* iceA2* allele in 63.6% (28 strains) of isolated strains from patients having NUD was associated statistically (*P* = 0.037) (Table 2).

### 3.3. Association between Age of Patients and* H. pylori *Genotypes

The 50.7% (38 strains) of* H. pylori* strains were isolated from patients under 40 years old (group 1) while the 49% (37 strains) were recovered from patients over 40 years old (group 2). Genotypes s1 and* s1m2* were more frequently found in patients belonging to group 2 while the genotypes s2 and* s2m2* were more often detected in group 1, both with a significant statistical association (*P* < 0.05).* H. pylori* strains isolated from patients over 40 years old were more frequent in those with PUD; between these two variables a statistical association was observed (*P* = 0.007) (Table 2).

### 3.4. *H. pylori* Strains Recovered from Different Gastric Regions

Fourteen* H. pylori* strains were isolated from two stomach regions, antrum and corpus of seven patients. In only one of these patients, a strain with identical genotype (*s1am1/cagA+/iceA2*) was found. However, in the majority of them, at least a variation in one of the investigated genes was observed. The variation percentages of the following genes,* cagA+*,* vacA* (s-region), and* iceA1,* were 57.1% (8/14 strains), 71.4% (10/14 strains), and 42.9% (6/14 strains), respectively (Table 3).

## 4. Discussion

In the current investigation the predominant types in Cuban* H. pylori* strains were the s1 (50/68 strains, 73.5%),* iceA2* (40/68 strains, 53.3%), and* cagA* gene (38/68 strains, 55.9%). Our results are in agreement with other developed studies in Cuba, Europe, and East Africa where a higher prevalence (70% or more) of the s1 subtype had been reported [9, 15, 16]. In contrast, an elevated frequency of strains belonging to s2 subtype was described in Jordan [17]. These data demonstrated the high genetic variability of strains in different countries. A low percentage of the strains (5%) harbouring both alleles s1 and s2 has been reported previously [18].

The current research is the first study developed in Cuba to analyze both the s1 variants of* vacA* gene and the gene* iceA* in* H. pylori* strains. Concerning previous research, the dominant* vacA* gene subtype in North and South America, Central Europe, and Australia was s1a [19] but in Portugal and Brazil was s1b [20, 21]. The same frequency of s1a and s1b subtypes, as it was observed in our study, is similar to reports from industrialised countries as France, Italy, USA, and Canada [22].

The existence of nontypeable* H. pylori* strains has been described previously, using similar primers [23]. In our investigation, two of the three sequenced strains were identified as s1a-s1b subtypes. Similar results have been informed by other authors, defining the consensus sequence as a recombinant of two originals with different subtypes each [24]. For m-region of the* H. pylori* strains, m1 and m2 subtypes were approximately equally prevalent as in Europe and Latin America [25].

The percentage of* vacA* genotype s1b/m1 in this study is similar to research developed in Mexico, Brazil, and Costa Rica [26]. However, in Japan and China, the most prevalent genotypes are s1c/m1 and s1c/m2, respectively [27].

In European, Venezuelan, and North American populations, only 60% of* H. pylori *isolates harboured* cagA* gene [16, 28], as what occurred in the current study. However, in Japan and Korea, the proportion of* cagA* + strains is usually over 90% [29]. In our investigation, the prevalence of* cagA* + strains is similar to the report of Torres [9] but is lower in comparison with another Cuban study (70%) [10].

The predominance of* iceA2* subtype is in agreement with reports from Colombia and USA [30]. Both subtypes of the* iceA* gene have been identified in Brazil, Malaysia, and Korea [31, 32].

Patients with* H. pylori* strains recovered from gastric antrum and corpus were infected with different* cagA*,* vacA,* and* iceA* genotypes. Also, multiple* H. pylori* genotypes were observed in a single biopsy specimen. Previous detailed molecular analysis has shown that each of the* H. pylori* strains contains only one* cagA* allele and each one of the s- and m-region subtypes of* vacA* gene. Therefore, it is an exact indicator for the presence of multiple strains of this organism if different genotypes are found [33]. The coexistence of more than one strain in the same individual may reflect the capacity of* H. pylori* to evolve genetic variations during the long-term colonization from childhood [3].

The absence of association between virulence genes explored and PUD could be influenced by the small number of patients studied with this pathology. Similar results have been described previously [3, 9, 10]. Moreover, the predominance of high virulence Cuban* H. pylori* strains in the group of patients with benign gastric diseases has unusual results in Western population. As it was reported in previous Cuban studies [9, 10], despite the presence of highly virulent* H. pylori*, the incidence of gastric cancer is lower in dyspeptic patients (gastric cancer death rate in Cuba: 7.5/100 000, http://files.sld.cu/dne/files/2014/05/anuario-2013-esp-e.pdf). Although this behavior has been observed in several previous studies [3, 7], it is probable that these findings suggest the action of environmental and host factors in Cuban patients. Further research studies to examine the role of host immunological factors might help to explain the different outcomes of* H. pylori*-induced disease in Cuban individuals.

We found an association between* H. pylori* strains harbouring the* iceA2* allele in patients with NUD. This behaviour has also been described in Europe, Saudi Arabia, and Turkey [34]. Several studies suggest an association of the* iceA1 *variant and PUD and between* iceA2* variants with gastritis [34, 35]. However, this association varies among populations; in Brazil, for instance,* iceA1* allele is associated with gastritis [31]. A recent meta-analysis confirms the relationship between the* iceA* allelic types and clinical outcomes [7].

The current investigation also showed statistical association of more virulent variants of* H. pylori* (s1 and* s1m2*) strains in the group of older patients. In Portugal and Tunisia, virulent strains have been detected in adult patients more frequently than children [36]. It has been described that* H. pylori* strains experiment recombination with others more virulent and better adapted strains to host changes, resulting in genotypes variable distribution between age groups [37, 38].

In summary, our results show a high prevalence of main virulence factors in Cuban isolates similar to that observed in other Western populations. In addition, we found strains with multiple genotypes, as it has been observed in countries with a high prevalence of* H. pylori* infection. Notably, a significant association was found among* iceA2* allele and NUD as well as strains with more virulent types and older patients. The* iceA* gene may be considered a useful marker in patients with gastroduodenal diseases. The relationship between* H. pylori* virulence factors and clinical outcomes in Cuban population is still unclear; therefore, further studies are required to determine the role of environmental and immunological factors.

## Figures and Tables

**Table 1:** Primers used in PCR for amplification of *cagA*, *vacA,* and *iceA* sequences and s1 region sequencing.

DNA amplified region	Primer	Primer sequence	PCR product	PCR program	Reference
		(5′-3′)	(bp)		
cagA	CAG-L	TGCTAAATTAGACAACTTGAGCGA	289	30 cycles (1 min at 95°C, 1 min at 50°C, and 1 min at 72°C)	[13]
	CAG-R	AATAATCAACAAACATCACGCCAT			

vacAs1a	SS1-F	GTCAGCATCACACCGCAAC	190	35 cycles (1 min at 95°C, 1 min at 56°C, and 1 min at 72°C)	[5]
vacAs1b	SS3-F	AGCGCCATACCGCAAGAG	187		
vacAs1c	SS1C-F	CTAGCTTTAGTGGGGATA	213		[14]
vacAs2	SS2-F	GCTAACACGCCAAATGATCC	199		[5]
	VA1-R^*^	CTGCTTGAATGCGCCAAAC			
vacA m1/m2	VAG-F	CAATCTGTCCAATCAAGCGAG	567/645^±^		[5]
	VAG-R	GCGTCAAAATAATTCCAAGG			

VacAs1	SIG-F	ATGGAAATACAACAAACACACCG	338		[8]
	SIG-R	CAACCTCCATCAATCTTACTGGA			
	VA1-F	ATGGAAATACAACAAACACAC	259		[5]

iceA1	iceA1-F	GTGTTTTTAACCAAAGTATC	246	30 cycles (1 min at 95°C, 1 min at 50°C, and 1 min at 72°C)	[8]
	iceA1-R	CTATAGCCATTATCTTTGCA			
iceA2	iceA2-F	GTTGGGTATATCACAATTTAT	229/334^¥^		
	iceA2-R	TTTCCCTATTTTCTAGTAGGT			

^*^Used as reverse primer with SS1-F, SS3-F, SS1C-F, SS2-F, and VA1-F. ^±^The size of the product is variable depending on the present subtype 567 bp for m1 and 645 pb for m2. ^¥^The primers yield a fragment of 229 or 334 bp depending on the presence of a repetitive sequence of 105 nucleotides codifying for 35 amino acids in some *iceA2* alleles.

**Table 2:** Association of *H. pylori* subtypes/genotypes with endoscopic findings and age of patients.

Genotypes		Clinical status			Age groups	
	NUD	PUD	*P* value	Group 1	Group 2	*P* value
	*n* = 44 (%)	*n* = 24 (%)		*n* = 35 (%)	*n* = 33 (%)	
*vacA *						
*s1 *	31 (70.5)	19 (79.2)	0.249	23 (65.7)	27 (81.8)	0.008^*^
*s2 *	11 (25.0)	3 (12.5)		12 (34.3)	2 (6.1)	
*s1-s2* ^±^	2 (4.5)	2 (8.3)		0 (0.0)	4 (12.1)	
*m1 *	20 (45.5)	15 (62.5)	0.179	17 (48.6)	18 (54.5)	0.622
*m2 *	24 (54.5)	9 (37.5)		18 (51.4)	15 (45.5)	
*s1m1 *	20 (45.5)	14 (58.4)	0.095	17 (48.6)	17 (51.5)	0.589
*s1m2 *	11 (25.0)	5 (20.8)	0.728	6 (17.1)	10 (30.4)	0.003^*^
*s2m1 *	0 (0.0)	1 (4.2)	—	0 (0.0)	1 (3.0)	—
*s2m2 *	11 (25.0)	2 (8.3)	0.395	12 (34.3)	1 (3.0)	0.016^*^
*s1-s2m2* ^±^	2 (4.5)	2 (8.3)		0 (0.0)	4 (12.1)	
*cagA *						
*cagA+ *	22 (50.0)	16 (66.7)	0.186	18 (51.4)	20 (60.6)	0.446
*cagA− *	22 (50.0)	8 (33.3)		17 (48.6)	13 (39.4)	
*iceA *						
*iceA1 *	14 (31.8)	11 (45.8)	0.037^*^	12 (34.3)	13 (39.4)	0.455
*iceA2 *	28 (63.6)	12 (50.0)		23 (65.7)	17 (51.5)	
*iceA1-iceA2* ^±^	2 (4.6)	1 (4.2)		0 (0.0)	3 (9.1)	
Clinical status						
NUD	—	—		28 (80)	16 (48.5)	0.007^*^
PUD	—	—		7 (20)	17 (51.5)	

NUD: non-peptic ulcer dyspepsia. PUD: peptic ulcer disease. Group 1: under 40 years old. Group 2: those within 40 years and older. ^*^Statistically significant (*P* < 0.05). ^±^Strains with multiple genotypes and seven strains recovered from gastric corpus were excluded from analysis. (—) This analysis is impossible to do.

**Table 3:** *H. pylori* strains recovered from gastric antrum and corpus of seven patients.

Patients	Stomach region	Endoscopic diagnostic	*vacA* gene subtypes		*cagA* gene presence^*^	*iceA* gene subtypes
73	A	NUD	s1b	*m1 *	−	*iceA2 *
	C		s1a, *s2 *	*m2 *	+	*iceA1, iceA2 *

71	A	NUD	*s2 *	*m2 *	+	*iceA2 *
	C		*s2 *	*m2 *	−	*iceA2 *

72	A	NUD	s1b	*m2 *	−	*iceA2 *
	C		*s2 *	*m2 *	+	*iceA1, iceA2 *

62	A	NUD	s1a	*m1 *	+	*iceA1, iceA2 *
	C		s1a, s1b	*m1 *	+	*iceA1, iceA2 *

69	A	NUD	*s2 *	*m2 *	−	*iceA1 *
	C		*s2 *	*m2 *	−	*iceA1, iceA2 *

78	A	NUD	*s2 *	*m2 *	+	*iceA2 *
	C		s1a, *s2 *	*m2 *	−	*iceA2 *

81	A	NUD	s1a	*m1 *	+	*iceA2 *
	C		s1a	*m1 *	+	*iceA2 *

A: gastric antrum. C: gastric corpus. NUD: non-peptic ulcer dyspepsia. ^*^Negative (−), *cagA* gene absent; positive (+), *cagA* gene present.
